# Tissue Doppler Imaging can be useful to distinguish pathological from physiological left ventricular hypertrophy: a study in master athletes and mild hypertensive subjects

**DOI:** 10.1186/1476-7120-7-48

**Published:** 2009-10-21

**Authors:** Giorgio Galanti, Loira Toncelli, Francesca Del Furia, Laura Stefani, Brunello Cappelli, Alessio De Luca, Maria Concetta Roberta Vono

**Affiliations:** 1 Postgraduate School of Sports Medicine-Sports Medicine Laboratory, University of Florence, Florence, Italy

## Abstract

**Background:**

Transthoracic echocardiography left ventricular wall thickness is often increased in master athletes and it results by intense physical training. Left Ventricular Hypertrophy can also be due to a constant pressure overload. Conventional Pulsed Wave (PW) Doppler analysis of diastolic function sometimes fails to distinguish physiological from pathological LVH.

The aim of this study is to evaluate the role of Pulsed Wave Tissue Doppler Imaging in differentiating pathological from physiological LVH in the middle-aged population.

**Methods:**

we selected a group of 80 master athletes, a group of 80 sedentary subjects with essential hypertension and an apparent normal diastolic function at standard PW Doppler analysis. The two groups were comparable for increased left ventricular wall thickness and mass index (134.4 ± 19.7 vs 134.5 ± 22.1 gr/m2; p > .05). Diastolic function indexes using the PW technique were in the normal range for both.

**Results:**

Pulsed Wave TDI study of diastolic function immediately distinguished the two groups. While in master athletes the diastolic TDI-derived parameters remained within normal range (E' 9.4 ± 3.1 cm/sec; E/E' 7.8 ± 2.1), in the hypertensive group these parameters were found to be constantly altered, with mean values and variation ranges always outside normal validated limits (E' 7.2 ± 2.4 cm/sec; E/E' 10.6 ± 3.2), and with E' and E/E' statistically different in the two groups (p < .001).

**Conclusion:**

Our study showed that the TDI technique can be an easy and validated method to assess diastolic function in differentiating normal from pseudonormal diastolic patterns and it can distinguish physiological from pathological LVH emphasizing the eligibility certification required by legal medical legislation as in Italy.

## Background

Left ventricular wall thickness and mass at transthoracic echocardiography are often increased in master athletes, a growing population of trained subjects observed in Sports Medicine. These modifications induced by intense physical training (athlete's heart) result from cardiac remodelling which is characterized by normal left ventricular performance and normal or increased capillary density with little or no fibrosis [1,2]. Increase in left ventricular mass may also be the consequence of a constant pressure overload, typical of the hypertensive state that is often present in this middle-aged population [3,4].

Two-dimensional echocardiography and conventional Pulsed Wave (PW) Doppler analysis of the diastolic function sometimes fails to distinguish the two aspects of left ventricular mass increase.

Pulse Wave-Tissue Doppler Imaging (PW-TDI) is a relatively new echocardiographic technique for analysing myocardial tissue movement [5-7]. Previous studies showed that assessment of mitral annulus movement with PW-TDI supplies an accurate evaluation of left ventricular diastolic function [8-10]. PW-TDI is less influenced by pre- and after-load variations than traditional PW Doppler [11]. Moreover, in subjects presenting an apparent "normal" pattern with traditional PW Doppler, PW-TDI allows to distinguish between normal and abnormal diastolic pattern [12].

Aim of this study is to evaluate the role of Pulsed Wave Tissue Doppler Imaging (PW-TDI) in differentiating normal from pathological diastolic patterns in the middle-aged population (athletes and hypertensives) with a mild-moderate LVM increase [13].

## Methods

From January 2004 to May 2006, 2573 master athletes (aged more than 40 years) and 1010 hypertensive patients were consecutively observed at our Non-invasive Cardiac Laboratory - Medicine Centre Sport, University of Florence. Among the master athletes we selected 80 regularly trained subjects (Group A), mean age 50 years, all practising agonistic competitive sports (cycling and running). They were matched for age, sex and body mass index with 80 sedentary hypertensive subjects (Group B) under pharmacological treatment and with a good control of the blood pressure daily profile. Inclusion criteria for both groups were mild-moderate LVM increase and an "apparent "normal diastolic pattern at echocardiographic evaluation. The reference limits defined in ASE guidelines for LVM are lower than those published in some previous echocardiographic studies and are virtually identical to those based on direct necropsy. Therefore, abnormal LVM increases were defined in mild, moderate and severe. Reference limits in men are 116 - 131 g/m^2 ^for mild, 132 - 148 g/m^2 ^for moderate and ≥149 g/m^2 ^for severe hypertrophy [13]. In current literature there is variability in fixing diastolic parameters normal range, mainly depending on the age. We adopted the Roldan 's classification [14,15] that defines the diastolic values matched for age with a 50 years cut-off (Tables 1 and 2).

**Table 1:** Normal Left Ventricle Doppler Filling Parameter

**Parameters**	**<50 yr**	**>50 yr**
Peak E (cm/s)	72 ± 14	62 ± 14
Peak A (cm/s)	40 ± 10	59 ± 14
E/A ratio	1.9 ± 0.6	1.1 ± 0.3
DT (msec)	179 ± 20	210 ± 36
IVRT (ms)	76 ± 11	90 ± 17

IVRT = isovolumetric relaxation time, DT = Deceleration Time.

**Table 2:** Types of pathological Doppler diastolic patterns

**Parameters**	**Abnormal relaxation**	**Pseudonormal**	**Restrictive**
Peak E (cm/sec)	↓	E>A	↑
Peak A (cm/sec)	↑	A<E	↓
E/A ratio	<1	>1 - <1,5	>1,5-2
DT (msec)	>220	normal	<160
IVRT (msec)	>100	normal	<70

Hypertensive subjects with an evident pathological diastolic pattern [16], as impaired myocardial relaxation or restrictive pattern were excluded.

For both groups other exclusion criteria were the presence of ischemic heart disease, major brady or tachy-arythmias, regional and/or global systolic ventricular dysfunction, pulmonary hypertension, pericardial and/or significant valvular heart disease and other major cardiovascular disease. In the athletes group, the presence of hypertension in medical history or check-in clinic, was considered an exclusion criteria. The study was approved by the Local Ethics Committee and all subjects enrolled gave their informed written consent.

### Basal echocardiographic evaluation

Echocardiographic studies were performed by four experienced ultrasonographer cardiologists using GE Vivid 7 equipped with 2.5 MHz and 3.3 MHz transducers. The following measurements were obtained in parasternal long-axis view, according to American Society of Echocardiography recommendations [17]: left ventricular (LV) end-systolic and end-diastolic diameters, measured at the base of heart just below mitral leaflet tips and interventricular septum, and posterior wall thickness. LV mass was calculated using the equation reported by Devereux et al [18]. In order to establish left ventricle geometric remodelling and to identify physiological hypertrophy by the traditional echocardiogram we also evaluated Relative Wall Thickness (RWT or h/r), that is the ratio between IVS and PW thickness (h) and LVED diameter (r). The range of the values obtained was corrected for age [19]. We also evaluated all other routine exam measurements (right chamber and left atrial dimension, pulmonary pressure, morphological and functional evaluation of cardiac valves).

### Traditional Doppler

**Figure 1:**
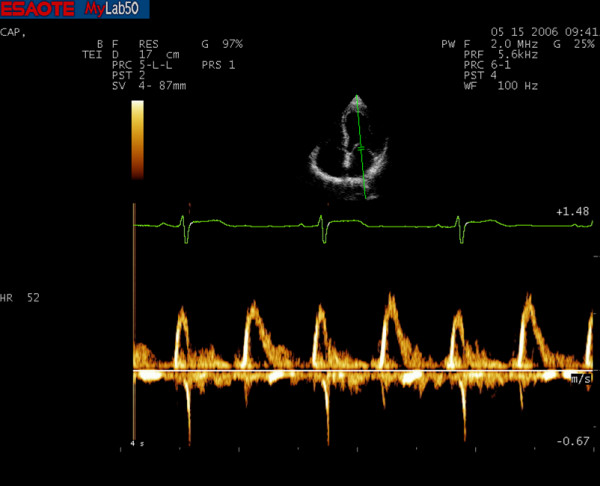
**Conventional PW Doppler spectrum of a master athlete's heart**.

**Figure 2:**
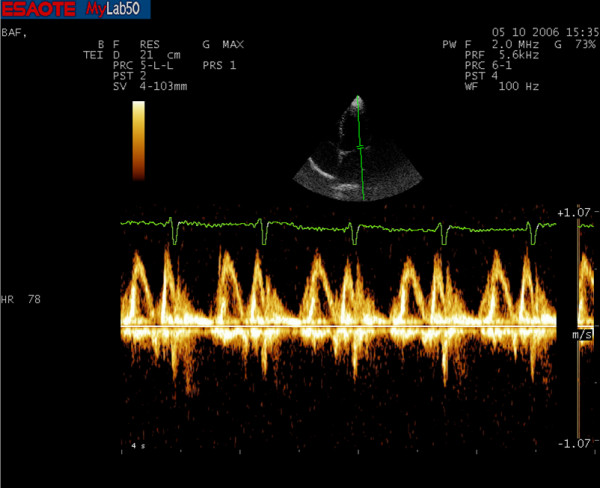
**Conventional PW Doppler spectrum of an hypertensive subject**.

Diastolic function was previously evaluated with traditional ultrasound PW Doppler of transmitral flow applied in a four-chamber apical view (Fig. 1 and 2). In this view a sample volume (2 mm) was placed between the tips of mitral leaflets. Early (E) and late (A) transmitral flow velocities, early to late peak velocity ratio (E/A) and E velocity deceleration time (DT) were analyzed. We also measured Isovolumetric Relaxation Time (IVRT) in five-chamber apical view with Continuous Wave (CW) Doppler.

There is a grading system for diastolic dysfunction based on the progression of disease patterns according a scale of I to IV grades. In the early stage of dysfunction, impaired relaxation of the left ventricle predominates. In "abnormal relaxation pattern" there is a low E velocity, prolongation of the deceleration time and increased A velocity (grade I). With disease progression, there is a "pseudonormal pattern" consist in a further increase of the E velocity and a shortening of deceleration time (grade II). In more advanced disease can occur a "restriction to filling pattern" characterized by increase of E velocity and shortening of DT (<140 to 150 ms; grade III).

### PW-TDI measurement

**Figure 3:**
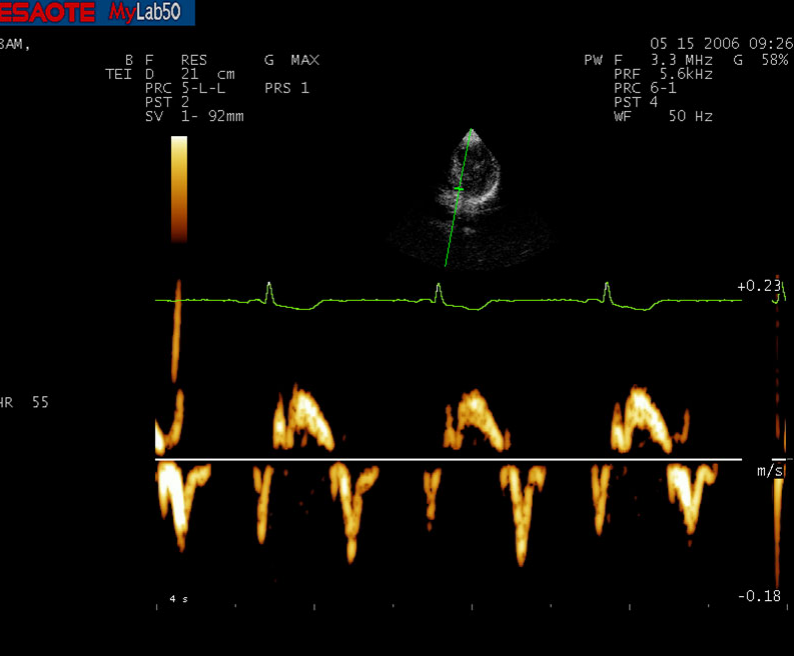
**PW-TDI spectrum of a master athlete's heart**.

**Figure 4:**
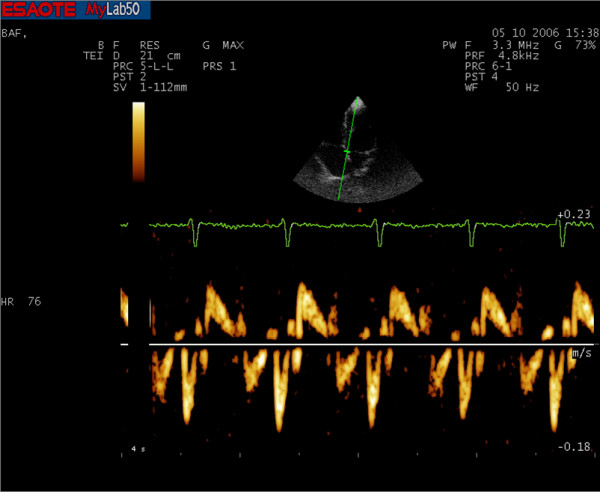
**PW-TDI spectrum of an athlete's heart**. It is possible already notice a reversal of Em and Am wave velocities than the master athletes.

Pulsed wave TDI was performed by activating TDI function in the same machine (Fig. 3 and 4). A sample volume (2 mm) was placed within mitral annulus septal myocardial wall, analyzing wall motion parallel to cursor orientation. We paid attention to have minimal angulation between the ultrasound beam and the plane of cardiac motion. Using pulsed wave spectral mode, filters and baseline were adjusted to a low velocity range; this technique allowed accurate determination of myocardial velocities. For the traditional Doppler, typically we used a "high pass" filter to eliminate the signals generated by low-speed movement of the walls and the gain was increased to amplify the signals of low intensity of red blood cells. For the TDI, however, we used a "low pass" filter to maintain the walls low speed signals and the gain was reduced to eliminate the low intensity signals of red blood cells. We set the velocity scale at about 20 cm/s above and below the zero-velocity baseline and used a 50 mm/s sweep speed. We obtained diastolic PW TDI Doppler indexes: early (E') and late (A') diastolic mitral annulus velocities. We then calculated mean parameters value acquired from the adiacent sites to obtain an accurate annular motion evaluation PW.

From the combination of traditional and TDI PW indexes, we then obtained another parameter (E/E' ratio), which was found in previous studies to be directly correlated with impaired diastolic function [20-23].

### Statistical analysis

Data were analysed by SPSS 13 and presented as means ± standard deviation and submitted to statistical analysis with Student's two-tailed unpaired test (p < .01).

## Results

There wasn't any significant difference in general data between two groups (Table 3).

**Table 3:** General characteristics

**Parameters**	**Athletes**	**Hypertensives**	**p**
Age	50.3 ± 10.0	51.7 ± 7.9	NS
Height	175 ± 6.32	172 ± 5.72	NS
Weight	75.5 ± 10.82	78.6 ± 2.99	NS
SBP	128 ± 5.13	134 ± 16.34	NS
DBP	76.81 ± 7.83	81.36 ± 8.09	NS
HR	60 ± 10	70 ± 5	NS

BP = blood pressure, HR = heart rate, SBP = systolic blood pressure, DBP = diastolic blood pressure.

### Echo General Data

Both groups showed a mild-moderate increase in LVM index (LVMI) without significant differences between the two groups (LVMI 134.4 ± 19.7 in athletes vs. 134.5 ± 22.1 in hypertensive subjects). Left Atrium (LA) and posterior wall were higher in hypertensive subjects. RWT in hypertensive ones was significantly higher than athletes and it was compatible with concentric hypertrophy. In both, left ventricular systolic function was normal (Table 4).

**Table 4:** Echocardiographic parameters

**Parameters**	**Athletes**	**Hypertensives**	**p**
LVDD (mm)	52.5 ± 3.4	49.8 ± 6.2	NS
LA (mm)	39.9 ± 3.3	38.2 ± 3.7	0.001
IVS(mm)	11.1 ± 0.9	11.5 ± 0.9	NS
LW (mm)	10.5 ± 1.0	11.0 ± 1.1	0.001
EF (%)	65.3 ± 3.38	63.5 ± 1.05	NS
D%	39.5 ± 3.69	36 ± 1.82	NS
Ventricular Mass Index (gr/m2)	134.4 ± 19.7	134.5 ± 22.1	NS
RWT (h/r)	0.41	0.45	0.001

LVDD = left ventricular diastolic diameter, LA = left atrium, IVS = interventricular septum, LW = Lateral wall, LVMI = left ventricular mass index, EF = ejection fraction (calculated by 2D), D% = shortening fraction, RWT = Relative Wall Thickness.

### Doppler Data

Results of diastolic function by traditional PW Doppler (Table 5) showed in both groups an "apparent normal diastolic pattern" without statistical differences in both groups in E velocities (71.2.2 ± 15.5 cm/sec in athletes and 72.3 ± 16.1 cm/sec in hypertensives), E/A ratio (1.2 ± 0.4 in athletes and 1.0 ± 0.4 in hypertensives) and DT (187.5 ± 40.4 in athletes and 197 ± 40.3 in hypertensives). A peak velocity was significantly different in the two groups (62.2 ± 15.5 in athletes and 72.5 ± 15.2 in hypertensives). IVRT value in hypertensives was significantly higher than in athletes (82.6 ± 12.2 msec in athletes and 95.6 ± 12.5 msec in hypertensive subjects p < .001), but according to Roldan's classification was still in normal range for age.

**Table 5:** Doppler Data

	**Athletes**	**Hypertensives**	**p**
E peak velocity	71.2 ± 15.5	72.3 ± 16.1	NS
A peak velocity	62.2 ± 15.5	72.5 ± 15.2	p < .001
E/A	1.2 ± 0.4	1.0 ± 0.4	NS
IVRT (msec)	82.6 ± 12.2	95.6 ± 12.5	p < .001
DT (msec)	187.5 ± 40.4	197 ± 40.3	NS
E/Em	7.8 ± 2.1	10.6 ± 3.2	p < .001

The PW-TDI study of diastolic function immediately differentiated the two groups. While in master athletes the diastolic TDI parameters were within the normal range, in entire hypertensives group E' was significantly lower (9.4 ± 3.1 cm/sec in athletes, 7.2 ± 2.4 cm/sec in hypertensives; p < .001); E/E' (7.8 ± 2.1 in athletes; 10.6 ± 3.2 in hypertensives) was significantly different too.

## Discussion

LV dimensions, volumes and wall thicknesses are echocardiographic measurements widely used in clinical practice and research. Methods for quantification of LV size and mass using 2-dimensional imaging have been validated and cross-checked 	with 3-dimensional data. Calculation of Relative Wall Thickness (RWT) permits categorization of an increase in LV mass as either concentric (RWT ≥ 0.42) or eccentric (RWT ≤ 0.42) hypertrophy and allows identification of concentric remodelling (normal LVM with increased RWT). In according to wall thickness values we can distinguish, in agreement with ASE recommendations, three groups in men: 11-13 mm indicates a mild LVH, 14-16 mm correspond to a moderate LVH and > 17 mm are values indicative of a severe LVH.

In this study we analysed two different groups of subjects (master athletes and hypertensive subjects) who were both characterized by a mild-moderate LVM and left ventricular wall increase and an apparently normal diastolic pattern at traditional PW Doppler analysis. The Valsalva maneuver reduces preload and can unmask a diastolic disfunction in subjects with an apparent normal diastolic pattern. In our study we didn't evaluate transmitral flow during a Valsalva maneuver in order to differentiate a normal from a pseudonormal pattern because of this method is mainly limited by induction of reflex tachycardia, which could result in a partial fusion of the E and A waves [24].

PW-TDI technique instead, in combination with the traditional PW Doppler analysis, was found to be useful in distinguishing physiological from pathological remodelling. In our study in fact all the 80 hypertensive subjects showed an apparent normal diastolic function by traditional PW Doppler but values out of the normal range by PW-TDI technique. This means that their diastolic pattern, analysed with PW Doppler, was a "pseudonormal pattern". The prevalence of "pseudonormal diastolic pattern" in general hypertensive population was about 7,9%. All hypertensive subjects of our study had this particular diastolic pattern but this is not surprising because we enrolled only hypertensive subjects with increased wall thickening and apparent normal diastolic function excluding those with an impaired relaxation by PW Doppler. Our findings confirmed the intrinsic limits of PW-Doppler-derived parameters to study diastolic function. TDI was useful to distinguish between normal and pseudonormal diastolic pattern.

E/E' ratio is an important parameter derived from a combination of traditional PW Doppler and PW-TDI. It's directly correlated with diastolic abnormality, in agreement with previous studies [25-27,1].

## Conclusion

Sometimes evaluating diastolic function only by PW-Doppler could be not so easy. Patterns of transmitral diastolic flow change mainly with the age and it also depends on the variation of pre- or after-load conditions. In certain subset of patients (sinus tachycardia, conduction system desease, arrhythmias), the diastolic function is more difficult to interpret. Sinus tachycardia and first-degree AV block can result in partial or complete fusion of E and A waves and DT may not be measurable. In atrial fibrillation the Doppler estimation is limited by the variability in cycle length and the absence of organized atrial activity.

Among different diastolic dysfunction patterns, the pseudonormal one is the hardest to interpret. The development of a new echocardiographic technique like PW Doppler TDI appears to be very important, helping us better understand and evaluate diastolic ventricular function.

LVM increase and LVH induced by a pathological state, such as hypertension, are associated to diastolic dysfunction that can occur even in early stages and are correlated with an increase in cardiovascular risk and mortality [28-30].

The evaluation of diastolic left ventricular function must be particularly accurate in middle-aged people, who are an ever-growing population involved in competitive sports. They had some systemic hypertension which, although in asymptomatic subjects, may lead to a diastolic dysfunction and an increase in mortality as compared to asymptomatic subjects with normal diastolic function [31,27,32].

The PW-TDI analysis is a more simple, inexpensive and immediate method to assess hypertrophic hearts than others methods like magnetic resonance. Therefore, it may become a useful instrument in distinguishing the physiological from the pathological LVH and increase of LVM, particularly when sport eligibility certification is required by legal medical legislation, as in Italy.

## Limitations

In our study we selected a particular subset of a middle-aged population; characterized by mild left ventricular increase mass and apparent normal diastolic pattern at conventional Doppler. The number of studied subjects was limited and the data should be confirmed on a large scale. Would be interesting in the future to compare our data with others methodologies (in e. DE-CMR), to confirm the hypothesis of the study.

## Competing interests

The authors declare that they have no competing interests.

## Authors' contributions

GG: concived of the study

LT: concived of the study, provided research, contributed to writing the article

FDF: provided research, provided statistical analysis, contributed to writing the article

LS: provided research

BC: provided research

ADL: provided research, contributed to translate and formatting article

MCRV: provided research.

All authors read and approved the final manuscript.
